# British Nursing Association

**Published:** 1888-03-31

**Authors:** 


### The Nursing Mirror.

Communications for this column should be addressed The Mirror, care of the Editor, 38, Old Jewry, London, E.C.

### BRITISH NURSING ASSOCIATION.

When reading The Hospital, which I take weekly, I find the "bye-laws" of the British Nurses Association, and as you suggest the "readers' views on the scheme," may I be allowed to say that I consider bye-law 14 to be perfectly absurd? Why should sisters of wards be asked to give the small sum of 2*s.* 6*d.* as a subscription, and matrons 10*s.* 6*d.*? I am perfectly aware that matrons and lady superintendents of large hospitals, who are receiving good salaries, are quite able to give a large sum; but what about matrons with salaries of £40 and under? Why, they cannot join, as they could not spare an annual subscription so large. I, for one, am now receiving a smaller salary than when ward sister, and because I am a matron I am asked to give treble the sister. Consequently I, with many others I know, cannot join the B.N.A. I consider it a great mistake that country matrons should have been kept out in the cold, never having been asked their opinion on the subject. It appears, up to the present time, that the Association is entirely in the hands of London matrons and lady superintendents. I enclose my card, and beg to remain, yours obediently, Matron.

